# Complete Closure of the Pulmonary Artery

**Published:** 1853-07

**Authors:** 


*Complete Closure of the Pulmonary Artery.—Cyanosis.*—Dr. Hare presented a specimen of this malformation, taken from a child who died at the age of nine months. During life, the limbs were colder than natural and of a dusky hue. Whenever the child coughed, or was moved much, he became distinctly blue. There was nothing unusual in the conformation of the chest, but close to the left nipple a purring tremor was detected, and at the apex of the heart a moderately loud systolic blowing murmur was audible. A *post-mortem* examination was made twenty-seven hours after death. There were about two drachms of fluid in the pericardium. The right auricle was much dilated and filled with blood. The foramen ovale was patulous, but extremely small. On cutting into the right ventricle, it was found that the carneæ columnæ were fused almost into one mass, so that this part of the right side of the heart was nearly solid. Towards the base of the ventricle, rudiments of the tricuspid valves were discernible, hanging into a cavity no larger than a moderate-sized pea. The pulmonary artery measured rather more than an inch in circumference at its middle, but the ventricular extremity terminated in a cul de sac, where there were some minute folds, probably rudiments of the pulmonary valves. At its upper extremity the vessel communicated with the aorta in the ductus arteriosus, the orifice of which was capable of admitting a crow-quill. The right and left pulmonary arteries were given off from the main trunk as usual. The left auricle was somewhat contracted, but the remaining structures of the heart were in their normal state. Dr. Hare remarked, that although instances of complete closure of the pulmonary artery were not rare, yet he thought the present specimen was almost unique, as exhibiting so contracted a foramen ovale in conjunction with that malformation.—*Lond. Med. Times and Gazette.*

